# Occam's Razor Versus Hickam's Dictum: A Case Report of Junctional Epidermolysis Bullosa and Lower Urinary Tract Infection

**DOI:** 10.7759/cureus.34117

**Published:** 2023-01-23

**Authors:** Abdur Rehman, Hassan Raza, Beenish Fatima Zia, Fatima Farahi, Meeran Asher Syed

**Affiliations:** 1 Cardiothoracic Surgery, Mayo Hospital, Lahore, PAK; 2 Medicine, Lahore Medical and Dental College, Lahore, PAK; 3 Medicine, Fatima Memorial Hospital College of Medicine and Dentistry, Lahore, PAK; 4 Clinical Research, Al-Shifa Trust Eye Hospital, Rawalpindi, PAK; 5 Internal Medicine, Fauji Foundation Hospital, Islamabad, PAK

**Keywords:** urinary tract infection, newborn and child health, oral mucosal lesions, junctional epidermolysis bullosa, hemorrhagic bullae

## Abstract

Epidermolysis bullosa (EB) is a rare heterogeneous group of diseases which typically presents with extensive blistering and mucocutaneous erosions. EB is mechanobullous in nature and thus commonly involves sites of trauma and friction. It is a painful and disfiguring disorder. The involvement of different internal organs and systems, such as respiratory, genitourinary, and gastrointestinal systems, has been reported in the literature depending on the type of EB. We report a case of junctional epidermolysis bullosa (JEB) with urogenital involvement in a female child in Pakistan. JEB is a rare subtype of EB which is transmitted in an autosomal recessive pattern of inheritance. It classically affects neonates. Diagnosis is established after clinical examination, and investigations are directed at the exploration of skin lesions such as histopathological and direct immunofluorescence studies. Management of patients is primarily supportive.

## Introduction

Epidermolysis bullosa (EB) is a rare inherited heterogeneous group of disorders marked by variable degrees of skin and mucosal fragility. EB is typically a painful, chronic, and disfiguring disorder. It is brought on by mutations that impair the skin's structural proteins leading to blister formation. It is unclear how common EB really is. According to the United States Epidermolysis Bullosa Registry, the overall incidence and prevalence of EB are 19.6 and 11.07 cases per million live births, respectively. The incidence and prevalence of junctional epidermolysis bullosa were found to be 2.68 and 0.49 cases per one million live births, respectively [1]. In the United States, the incidence rate is 20 per million births [2]. The lesions frequently manifest as thick blisters that may later rupture and leave scars. They might first arise after birth or at any point up until early adulthood. Children's hands, feet, and diaper areas are the most common places for blisters to develop (at the sites of trauma or pressure), although they can also develop in the mouth, gastrointestinal system, or genitalia. Co-occurring disorders in EB patients may include fused digits and clubbed hands or feet [3].

EB can be divided into the following four types (based on different sites of blister formation within the skin structure): 1) epidermolysis bullosa simplex (EBS): which causes blisters in the epidermis, 2) dystrophic epidermolysis bullosa (DEB): which causes blisters at the level of lamina densa and upper dermis, 3) junctional epidermolysis bullosa (JEB): which causes blisters in the epidermal-dermal junction, and 4) Kindler syndrome: which causes blisters at various levels of the skin [4]. Skin blistering is a common feature of all kinds of EB, despite the fact that clinical presentation or the degree of ulceration may differ according to the varied phenotypes. Clinical observation can frequently lead to a provisional diagnosis, but histological examination of a skin sample yields the most conclusive results in the diagnosis of EB. Transmission electron microscopy, antigenic mapping, and immunofluorescence (IF) are essential diagnostic techniques for confirming and identifying a specific EB subtype [5]. The subtype of the illness can have an impact on its prognosis.

## Case presentation

A two-year-old female presented to the paediatrics outpatient department by her parents with a complaint of urinary retention for two days. The mother of the child further emphasized the painful micturition whenever the child tried to void. She further added that a very small amount of urine that the child used to pass was foul-smelling. The child had blisters and granulation tissue all over her body. Her mother explained that she had those skin lesions since the time of birth and that she was a clinically diagnosed case of epidermolysis bullosa (EB). But because of the non-availability of required diagnostic procedures, the exact type and subtype of EB was not explored.

Upon abdominal examination, a painful bladder was noted. A complete blood picture showed an increase in WBC count (16.5x10^9^/L) and a decrease in haemoglobin level (4.6 g/dl). Peripheral smear of blood showed RBC morphology as anisopoikilocytosis with hypochromic microcytic red cells, WBC morphology as leukocytosis with left shift, and platelets morphology as reactive thrombocytosis. A provisional diagnosis of lower urinary tract infection (UTI) was made. Urine culture proved *Escherichia coli* (*E. coli*) to be the causative agent for the infection.

**Figure 1:**
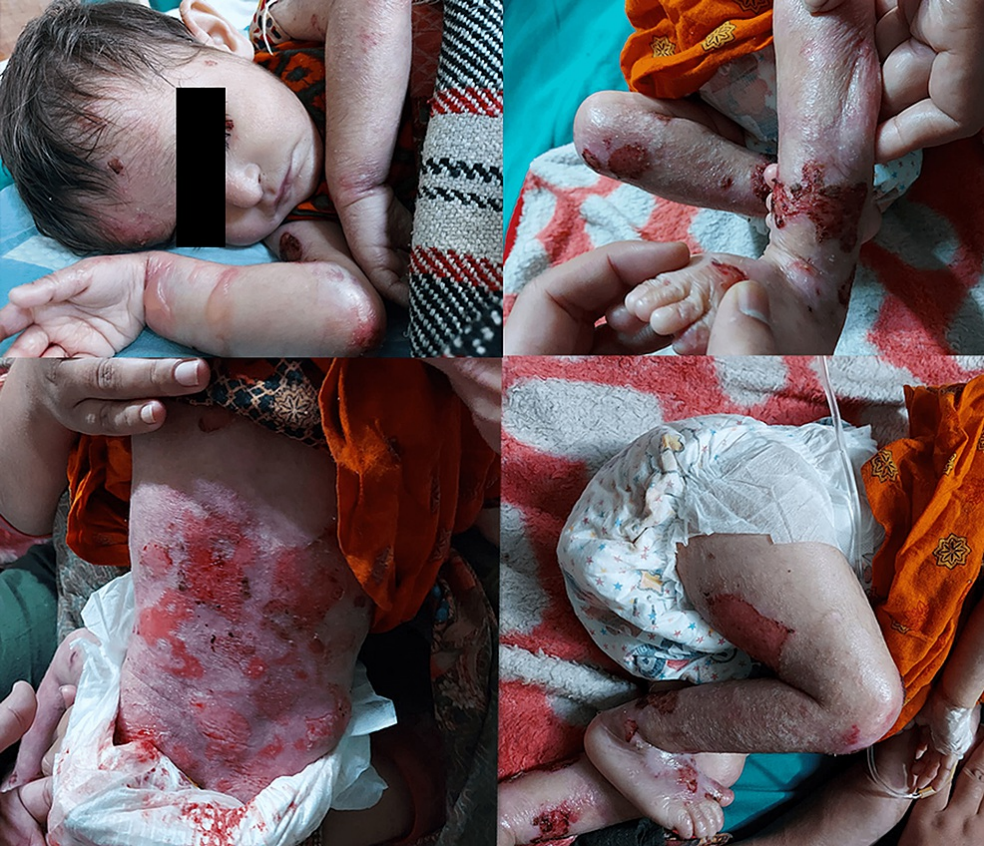
Bullae (on right forearm; top left), granulation tissues (on left ankle; top right), rashes (on back; bottom left), and several vesicles (bottom right).

In the context of EB, mucosal involvement caused a stricture in the lower urinary tract which was suspected to be the root cause of the infection. Hence, a retrograde urethrogram (RUG) was done. Interestingly, the investigation showed no stricture. So poor toilet and hygiene habits were devised to be the cause of lower urinary tract infection. Haemorrhagic oozing from the skin lesions was observed. On physical examination, several vesicles, bullae, rashes, and granulation tissues on the face, arms, trunk, legs, and ankles were documented (Figure 1). The parents were of consanguineous marriage, and the siblings of the patient did not show any features of EB.

The detailed history of skin lesions revealed that multiple blisters on the skin of the dorsal aspect of the left ankle were observed right on the day she was born. Three days later, she developed clear fluid-filled lesions which started from her hands, and within two days, they progressed to the whole body including both feet. They ruptured spontaneously, and lesions acquired the same size as the blisters. A permanent formation of vesicles was observed which have a healing erosive surface with a bright red base. These lesions took about a week to heal. There was also ulceration and blistering in the oral cavity which caused difficulty in feeding. It remained for one year and then settled. For one year, lesions were only limited to hands and feet, but later, they involved other areas as well. There was a relapsing pattern.

**Figure 2:**
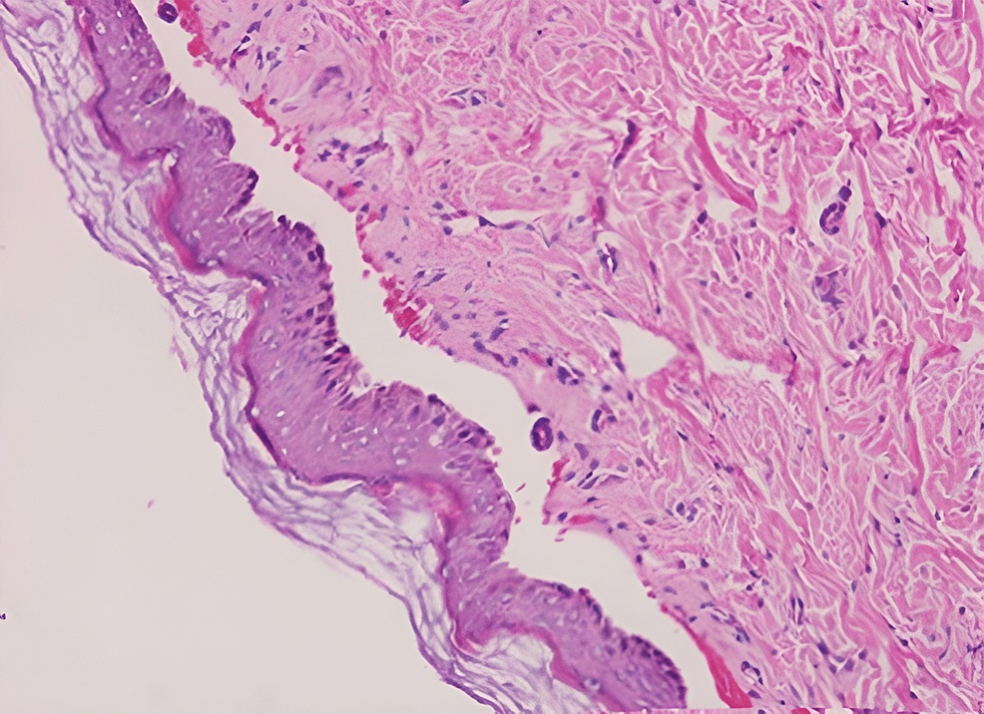
Histopathological slide (haematoxylin and eosin stain) showing clefting and blister formation at the junction of dermis and epidermis.

**Figure 3:**
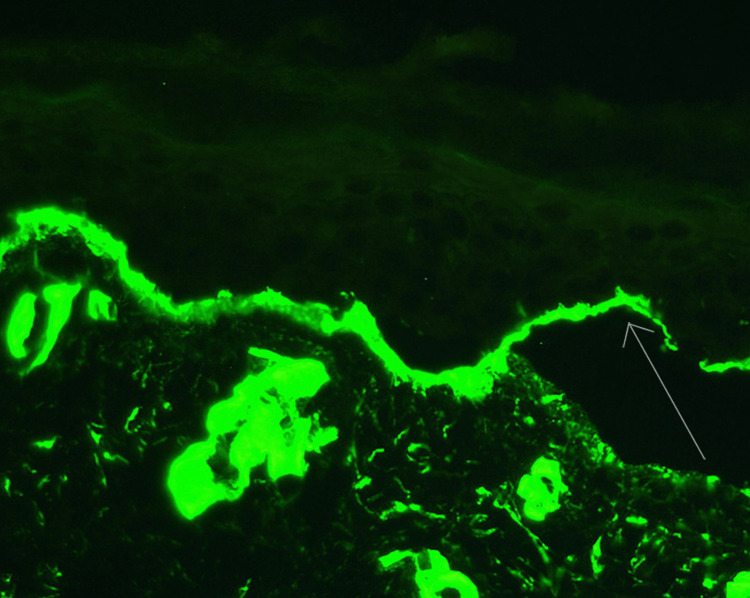
Direct immunofluorescence mapping with fluorescence antisera against type IV collagen (localized on the basal lamina) indicates a positive linear immunofluorescence at the base of the blister (arrow).

Histopathological investigations (of bullous lesion on right forearm) revealed sub-epidermal blistering with no inflammatory cells. The roof of the blister comprised a full-thickness epidermis (Figure 2). Direct immunofluorescence mapping with fluorescence antisera against type IV collagen (localized on the basal lamina) indicates a positive linear immunofluorescence at the base of the blister. This allocation of type IV collagen in the blister shows a junctional cleavage (Figure 3).

Based on these findings, a diagnosis of JEB was established. She has been receiving supportive care. Fusidic acid cream and liquid paraffin were recommended to apply on the lesions.

## Discussion

A spectrum of connective tissue illnesses known as epidermolysis bullosa can affect many different organs, including the kidneys, intestines, heart, and skin. It causes tissue fragility, especially near the skin's surface [1,6]. It is an inherited connective tissue disease that is painful, recurring, disfiguring, and persistent.

When a newborn exhibits blisters, a wide range of differential diagnoses are available, including infectious conditions like bullous impetigo, toxic epidermal necrolysis, staphylococcal scalded skin syndrome, and immunologic conditions like pemphigus or bullous pemphigoid, as well as hereditary conditions like EB. Differential diagnosis is based on bacterial culture, direct immunofluorescence, histology, and clinical symptoms. Blisters that form inside the lamina lucida are a hallmark of the autosomal recessive condition JEB. Clinical manifestations are typically used to differentiate between subtypes, which include the generalised gravis (Herlitz) type, generalised mitis (non-Herlitz) type, cicatricial, acral, inverse, and progressive [7]. Based on the clinical symptomatology, histological examination, and immunofluorescence studies, this patient most likely had Herlitz-type JEB, which manifested as widespread blistering.

Since all EB subtypes show up as subepidermal splits under light microscopy, histopathology is typically not very helpful in identifying a specific subtype of EB [8]. EBS exhibits an intraepidermal separation at the level of the basal cells in terms of ultrastructure; meanwhile, JEB and DEB exhibit subepidermal separations through the lamina lucida or beneath the lamina densa, respectively [9]. Immunofluorescence mapping allows for the quick and accurate detection of the degree of tissue separation. This approach, which is based on immunofluorescence staining, links the degree of cleavage to particular structural protein markers that are strongly expressed in both healthy skin and the skin of EB patients [10]. Numerous macromolecules that are necessary for the cohesiveness of the skin and its resistance to shearing forces have come to light in recent studies. It has expanded our understanding of the genetic changes and distinctive ultrastructural morphology that cause skin blistering [5].

The reported case has a significant clinical interest because of the severity and rarity of EB in Pakistan. The patient's quality of life may be significantly impacted by the severe form because it can be tough and challenging to manage. Patients with EB have few alternatives for treatment. The main goals of treatment are to safeguard the skin, lessen blistering, avoid problems, and encourage healing. They require specialised continuous treatment, regular dressing changes, and a multidisciplinary approach that includes a dermatologist, medical geneticist, internist, anaesthetist, paediatrician, neonatologist, pathologist, pain specialist, trained nurses, and psychiatrist or psychologist. The team may also include a dentist, gastroenterologist, ophthalmologist, otolaryngologist, and endocrinologist. Follow-up is necessary forever [11]. Prevention of trauma, meticulous wound care, nutritional support, and infection control are the main components of the management of EB patients. When blistering and scarring result in abnormalities, surgical procedures are recommended [12]. Since EB is a genetic illness, the underlying molecular defect cannot be corrected by medication [13]. The multivalent wound care ointment offers a promising solution for the efficient management of moisture and bioburden in EB patients. Jojoba oil has been found in studies to help active substances penetrate the skin more easily [14].

## Conclusions

This case is particularly interesting because it emphasizes the importance of Hickam’s dictum, especially when it comes to the exploration and understanding of rare diseases. In the given case, the involved team initially bludgeoned the diagnostic possibilities into Occam’s razor. Said otherwise, it was easy to explain urinary symptoms under the umbrella of EB, rather than considering it to be a separate unrelated entity. A patient can have multiple pathologies contributing to the same presentation. Clinicians, therefore, should keep an open mind regarding the possibility of coexisting illnesses, once the first diagnosis has been confirmed.

The subtype of epidermolysis bullosa must be correctly diagnosed to offer genetic counselling and prognostic information. Since there is currently no treatment for epidermolysis bullosa, avoiding trauma and promoting rapid wound healing are the cornerstones of management. At birth, the JEB has a hallmark of mechanical fragility which manifests as severe skin blistering accompanied by crusting and erosions. There are numerous challenges in treating EB patients. Since the precise causes of EB are yet unknown, compassionate therapy using a multidisciplinary approach is recommended.
